# Research Progress on Sepsis Diagnosis and Monitoring Based on Omics Technologies: A Review

**DOI:** 10.3390/diagnostics15222887

**Published:** 2025-11-14

**Authors:** Xinhao Jin, Hongjie Shen, Pengmin Zhou, Jie Yang, Suibi Yang, Hongying Ni, Yuetian Yu, Zhongheng Zhang

**Affiliations:** 1 Department of Critical Care Medicine, Sir Run Run Shaw Hospital, Zhejiang University School of Medicine, Hangzhou 310016, China; jinxinhao@zju.edu.cn; 2 Department of Emergency Medicine, Sir Run Run Shaw Hospital, Zhejiang University School of Medicine, Hangzhou 310016, China; hongjie.srrsh@gmail.com (H.S.); pmzhou@126.com (P.Z.); 22218206@zju.edu.cn (J.Y.); 22418554@zju.edu.cn (S.Y.); 3 Department of Intensive Care Unit, Affiliated Jinhua Hospital, Zhejiang University School of Medicine, Jinhua 321000, China; nihongying2@163.com; 4 Department of Critical Care Medicine, Renji Hospital, School of Medicine, Shanghai Jiao Tong University, Shanghai 200001, China; 5 Key Laboratory of Precision Medicine in Diagnosis and Monitoring Research of Zhejiang Province, Sir Run Run Shaw Hospital, Zhejiang University School of Medicine, Hangzhou 310016, China; 6 School of Medicine, Shaoxing University, Shaoxing 312000, China; 7 Longquan Industrial Innovation Research Institute, Lishui 323700, China

**Keywords:** sepsis, omics, multi-omics, biomarker discovery, CMAISE database

## Abstract

Sepsis poses a significant global health burden, with millions of cases and high mortality rates annually, largely due to challenges in early diagnosis and monitoring. Traditional methods, reliant on nonspecific clinical manifestations and limited biomarkers like C-reactive protein and procalcitonin, often fail to distinguish infection from non-infectious inflammation or capture disease heterogeneity. This review synthesizes recent progress in omics technologies—genomics, transcriptomics, proteomics, and metabolomics—for advancing sepsis management. Genomics, via metagenomic next-generation sequencing, enables rapid pathogen identification and genetic variant analysis for susceptibility and prognosis. Transcriptomics reveals molecular subtypes and immune dynamics through RNA sequencing and single-cell approaches. Proteomics and metabolomics uncover protein and metabolite profiles linked to immune imbalance, organ damage, and metabolic disorders. Multi-omics integration, enhanced by artificial intelligence and machine learning, facilitates biomarker discovery, patient stratification, and predictive modeling, bridging laboratory findings to bedside applications like rapid diagnostic tools and clinical decision support systems. Despite advancements, challenges including data heterogeneity, high costs, and ethical concerns persist. Future directions emphasize single-cell and spatial omics, AI-driven personalization, and ethical frameworks to transform sepsis care from reactive to proactive, ultimately improving outcomes.

## 1. Introduction

### 1.1. Background and Current Status of Sepsis

Sepsis is life-threatening organ dysfunction due to a dysregulated host response to infection [1]. According to the Sepsis-3 definition from The Third International Consensus Definitions for Sepsis and Septic Shock in 2016, it emphasizes infection-induced organ dysfunction over systemic inflammatory response syndrome (SIRS). The core pathogenesis of sepsis is biphasic immune dysregulation—early hyperinflammation and late immunosuppression [2]. Pathogen-associated molecular patterns (PAMPs, such as LPS from Gram-negative bacteria) and damage-associated molecular patterns (DAMPs) activate the NF-κB signaling pathway through Toll-like receptors (TLR4/2), leading to a cytokine storm of pro-inflammatory factors like TNF-α and IL-6. Simultaneously, the NLRP3 inflammasome is activated, promoting caspase-1-dependent cleavage of GSDMD protein, inducing pyroptosis and releasing IL-1β/IL-18, exacerbating tissue damage [3,4,5,6]. Microcirculatory dysfunction further worsens organ function: disruption of the endothelial glycocalyx and DNA-PKcs-mediated depolymerization of the cytoskeleton lead to microthrombus formation, aggravating hypoxia and metabolic disorders [7]. In the later stages, immunosuppression appears, characterized by weakened pathogen clearance and increased risk of secondary infections [8].

According to data from the Global Burden of Disease Study 2020, there were approximately 48.9 million cases of sepsis worldwide in 2017 (95% UI: 38.9–62.9 million), resulting in 11 million deaths (95% UI: 10.1–12 million), accounting for 19.7% of global deaths [9]. Mortality has declined in high-income countries due to improved early diagnosis and treatment, but remains high in resource-limited areas. The long-term prognosis of sepsis is also concerning, as surviving patients may face persistent inflammation, immunosuppression, and organ dysfunction, leading to increased post-discharge mortality risk [10].

### 1.2. Challenges in Sepsis Diagnosis and Monitoring

The diagnosis and monitoring of sepsis currently face numerous challenges [11,12]. First, clinical manifestations of sepsis (such as fever, tachycardia, tachypnea, or altered mental status) are nonspecific and overlap with other inflammatory diseases (such as trauma or non-infectious inflammation), increasing the risk of misdiagnosis [13]. Second, laboratory indicators for sepsis lack specificity, making early diagnosis difficult. For example, traditional inflammatory markers like white blood cell count, C-reactive protein (CRP), and procalcitonin (PCT) are widely used but have limited sensitivity and specificity, struggling to accurately distinguish between infectious and non-infectious inflammation [14,15]. Third, rapid identification of pathogens remains a major challenge; traditional culture methods are slow with low positivity, while metagenomic next-generation sequencing (mNGS) improves detection speed and rate but is costly and complex, and it is difficult to distinguish between infection, colonization, or contamination [16]. Additionally, the heterogeneity of sepsis patient populations (such as differences in underlying diseases and immune status) and dynamic changes mean that most existing studies focus on single time points, failing to capture dynamic features and limiting the clinical utility of biomarkers [17].

**Figure 1:**
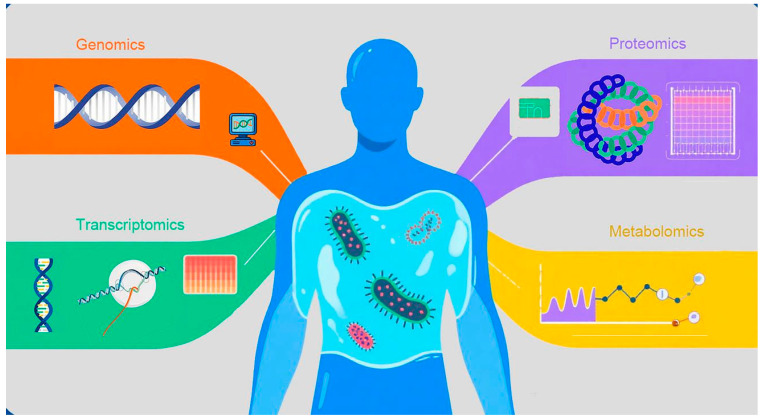
Fundamental Concepts of Omics Technologies and Their Dimensions in Sepsis Molecular Subtyping. This figure visually illustrates how different omics technologies (genomics, transcriptomics, proteomics, and metabolomics) provide data from distinct molecular dimensions. Each technology represents a critical aspect of the disease state, collectively forming a multi-dimensional molecular portrait. These dimensions serve as the foundation for identifying patient molecular subtypes and assessing individualized differences, thereby helping readers intuitively establish the conceptual basis of omics in precision medicine.

Omics technologies—genomics, transcriptomics, proteomics, and metabolomics—analyze DNA, RNA, proteins, and metabolites, respectively [18,19,20,21,22], providing microscopic, dynamic insights into sepsis pathophysiology (Figure 1). The central thesis of this review is to demonstrate how advances in omics technologies address sepsis heterogeneity, enabling a paradigm shift from “one-size-fits-all” treatment to precision and personalized management. Multi-omics integration comprehensively reveals mechanisms, identifies specific biomarkers, and supports precise diagnosis and individualized therapy [23,24]. Combined with AI and machine learning, they optimize biomarker screening and advance sepsis diagnosis and monitoring.

## 2. Overview of Omics Technologies in Sepsis

“Omics” is a field of biological research whose core principle is the systematic, holistic study of all molecules of the same class within an organism, rather than examining individual molecules in isolation. It primarily encompasses several branches: genomics (the study of all DNA sequences), transcriptomics (the study of all RNA transcripts), proteomics (the study of the entire set of proteins), and metabolomics (the study of all small-molecule metabolites).

The omics research workflow follows a clear pipeline designed to transform raw data into clinically actionable signals: first, high-throughput technologies (e.g., sequencing, mass spectrometry) are used to measure biological samples, generating massive raw datasets. Next, bioinformatics analysis performs quality control, molecular identification, and abundance quantification; differential analysis across groups (e.g., healthy vs. diseased) identifies key molecules and elucidates their functional roles. Finally, machine learning and integrative methods convert these molecular features into practical clinical applications—such as discovering biomarkers for early diagnosis, enabling precise molecular subtyping to guide therapy, or building predictive models to support clinical decision-making—ultimately advancing precision medicine.

### 2.1. Genomics

Genomics has revolutionized sepsis diagnosis and monitoring. Traditional methods (symptoms + limited markers like temperature, WBC, CRP, PCT) hinder early precise diagnosis. mNGS sequences all nucleic acids in samples (e.g., blood, BALF), rapidly identifying bacteria, viruses, and fungi within hours, boosting efficiency, accuracy and pathogen spectrum [25,26]. Furthermore, genomics has accelerated the discovery of diagnostic and prognostic biomarkers for sepsis. Tong et al. used Short time-series expression miner (STEM) analysis and the least absolute shrinkage and selection operator (LASSO) model to screen 9 key dysregulated genes (CHPT1, CPEB4, DNAJC3, etc.) from differentially expressed genes, validated in 69 clinical samples showing high expression in sepsis patients, significantly correlated with immune cell infiltration (e.g., increased neutrophil and endothelial cell infiltration, decreased T cell and NK cell infiltration) [27]. These key dysregulated genes and their correlation with immune cell dynamics suggest they are promising candidates for novel diagnostic biomarkers and potential therapeutic targets for precision medicine in sepsis. Fan et al. utilized machine learning algorithms (random forests, support vector machine, etc.) from multiple GEO datasets to identify 15 sepsis shock diagnostic gene markers (e.g., CLEC5A, DUSP3, ARHGEF18), where ARHGEF18 and FCER1A were significantly related to patient survival; the RF model’s diagnostic AUC reached 0.993, demonstrating excellent clinical potential [28]. This demonstrates that these markers are excellent candidates for a highly accurate diagnostic panel to swiftly identify septic shock, while the survival-related genes (ARHGEF18 and FCER1A) offer critical prognostic information for patient risk stratification and personalized therapy.

In a cross-disease association study, Yang et al. used WGCNA and machine learning to identify common hub genes ARG1 and HP in severe acute pancreatitis (SAP) and sepsis, linking them to inflammation via NLR signaling pathways; their diagnostic model achieved a perfect AUC of 1.000 [29]. While this provides promising new targets for mechanistic exploration, the reliability of these genes as clinical biomarkers must be rigorously verified in independent, prospective clinical cohorts and must demonstrate quantified impact on patient-important outcomes.

In studies identifying genetic variants’ impact on sepsis susceptibility and prognosis, Davenport et al. used comprehensive genomic approaches and expression quantitative trait loci (eQTL) analysis to discover 3795 cis-eQTLs and 171 trans-eQTLs in sepsis patients, involving key immune regulatory genes like TLR4 and TNF; these eQTLs are enriched in endotoxin-induced epigenetic marker regions, suggesting that genetic variants may influence host responses to infection through gene expression regulation. For example, eQTLs of the mTOR gene were significant only in the SRS1 subtype, indicating subtype-specific genetic regulation [30]. While this data illuminates the genetic basis of differential host response, clinicians will never screen for thousands of eQTLs. The eventual goal of this early-stage genomic research is to refine these findings down to a small, actionable panel of core genetic variants/genes for prognostic prediction, genetic subtyping, and guiding personalized, targeted therapies in sepsis.

Genomics also provides new ideas for individualized sepsis treatment. Russell noted that genetic variants in thrombomodulin (TM) and endothelial protein C receptor (EPCR) are associated with mortality in sepsis patients and may predict responses to recombinant human TM or activated protein C (APC) therapy. Additionally, the efficacy of common drugs like epinephrine, vasopressin, and corticosteroids may be regulated by polymorphisms in genes such as ADRB2, AVPR1a, and NR3C1, providing molecular basis for optimizing treatment regimens [31].

Moreover, functional enrichment analysis shows that sepsis differentially expressed genes are mainly enriched in metabolic pathways, inflammatory pathways, and immune regulatory pathways [27,28,29], providing mechanistic foundations for targeted therapy. These genomics-based monitoring approaches provide strong support for clinicians to diagnose sepsis timely, monitor its progression, adjust treatment strategies, and improve patient outcomes.

### 2.2. Transcriptomics

Transcriptomics has advanced rapidly, using high-throughput sequencing (e.g., RNA-seq, scRNA-seq) to capture spatiotemporal gene expression differences, revealing regulatory networks, pathway dynamics, and disease-specific features. Compared to traditional gene expression analysis, transcriptomics can more systematically parse molecular mechanisms of biological processes, especially in studies of disease heterogeneity, offering significant advantages. This provides key tools for parsing sepsis molecular features, identifying biomarkers, monitoring sepsis progression, and guiding individualized treatment.

Transcriptomic technologies have greatly aided sepsis subtyping. Several sepsis subtypes have been identified through transcriptomics. Wong et al. classified pediatric sepsis into three subtypes, with subtype A showing higher mortality due to suppressed adaptive immunity and glucocorticoid receptor signaling [32]. Identifying Subtype A patients early allows for accelerated, targeted interventions, potentially including immunomodulatory therapies. Davenport et al. defined the immunosuppressive SRS1 profile (T cell exhaustion, HLA II downregulation) [30], and Scicluna et al. identified four molecular endotypes of sepsis (Mars 1–4), highlighting BPGM and TAP2 as important biomarkers for the worst prognosis Mars1 subtype [33]. Sweeney et al. identified and validated three stable molecular endotypes of sepsis through integrated analysis of whole-blood transcriptomic data from 700 bacterial sepsis patients across 14 datasets in 8 countries [34]. These molecular signatures can be quickly integrated into clinical panels to predict mortality, assess immunosuppression status, and guide individualized treatment plans. Furthermore, the conservation of endotypes across pathogen types (such as in COVID-19 patients) [35] validates these signatures for personalized therapy selection.

While Bulk RNA-seq measures average signals, single-cell RNA Sequencing (scRNA-seq) provides single-cell resolution to overcome averaging and heterogeneity challenges. Reyes et al. performed scRNA-seq on peripheral blood mononuclear cells from sepsis patients and controls, identifying a significantly expanded CD14 monocyte subtype MS1 (expressing RETN, ALOX5AP, and IL1R2) that effectively distinguished sepsis from controls and non-infectious inflammation [36]. This represents a significant, high-resolution marker discovery (Level II–III evidence) but its clinical value remains diagnostic, lacking sufficient prospective validation to demonstrate quantified guidance on patient-important outcomes. Zhang et al., in an E. coli-induced sepsis mouse model, demonstrated that the Mono2 subset increased in lethal models, highly expressing TNF and NF-κB pathway genes. They further used public human data to validate diagnostic and prognostic genes, demonstrating cross-species conservation and offering new mechanistic insights into progression [37]. While this study provides strong mechanistic insight into lethal disease trajectories (Level IV evidence), its direct clinical translatability is constrained by the mouse model, necessitating validation of dynamic predictions in human prospective cohorts.

Additionally, the regulatory role of non-coding RNAs in sepsis has gained attention in recent years. Cheng et al. constructed a glycolysis-related ceRNA network (SNHG17/miR-214-3p/IER3) for sepsis-induced cardiomyopathy (SIC) by integrating transcriptomic data from GEO databases; IER3 expression was negatively correlated with oxygenation index, miR-214-3p negatively correlated with NT-proBNP, and the network’s diagnostic efficacy AUC reached 0.942, providing a new explanation for SIC metabolic disorders [38].

In summary, transcriptomics has achieved significant results in sepsis diagnosis and monitoring; through subtyping and diagnostic model construction, it enables more accurate diagnosis of sepsis or its complications; in monitoring, it can be used for immune status monitoring and guiding clinical medication and treatment strategies.

### 2.3. Proteomics

Proteomics studies the entire proteome (expression, modifications, interactions, functions) in organisms, cells, tissues, or fluids using separation (two-dimensional gel electrophoresis by isoelectric point/molecular weight; liquid chromatography) and identification (mass spectrometry via *m*/*z* of digested peptides) techniques, offering high resolution, efficiency, sensitivity, and accuracy. Recent advances in affinity platforms (SOMAscan, Olink) have enhanced coverage and precision, making proteomics vital for clinical sepsis research [39,40].

Proteomics provides direct evidence for elucidating immune imbalance, lipid metabolism disorders, and organ damage mechanisms in sepsis. In immune regulation, top-down and bottom-up proteomics were used by Dubois et al. to analyze S100A8/S100A9 complexes in septic shock plasma [41]. They identified specific proteoforms (such as mono-oxidized S100A8 and truncated acetylated S100A9) that were significantly elevated in non-survivors. These findings translate into precise prognostic markers, indicating that specific structural changes in inflammatory proteins, driven by oxidative stress, are critical drivers of poor patient outcomes. In lipid metabolism disorders, Mi et al.’s large-scale plasma proteomics analysis of sepsis patients showed that disease severity was linked to lipid biology-related proteins like APOA1 and PON1 [42], a theme also noted in other reviews [43]. The dysregulation of these key lipid transport proteins highlights the profound metabolic collapse in sepsis and suggests potential therapeutic avenues targeting lipid pathways to restore homeostasis. Furthermore, non-invasive urine biomarkers are crucial for monitoring organ damage. Urine proteomics has revealed specific molecular features of sepsis-associated acute kidney injury (AKI). Stanaway et al. identified two distinct AKI subphenotypes AKI-SP1 and AKI-SP2 with different urine protein profiles [44]. AKI-SP1 proteins were associated with tissue repair and regeneration, while AKI-SP2 proteins involved immune-inflammatory responses and were linked to bacteremia. This subphenotyping ability provides promising insights for improving early identification, predicting the eventual need for RRT, and implementing or developing targeted therapeutic strategies tailored to the patient’s specific kidney damage mechanism (repair vs. inflammatory) [45].

Additionally, for plasma biomarkers, Dubois et al.’s mass spectrometry method confirmed S100A8/S100A9 as important prognostic indicators for monitoring septic shock patients. Due to its antibody-free, simple, reliable, and easily standardized detection process, it can be quickly replicated in hospital laboratories, helping clinicians identify high-risk patients early and decide on high-risk or high-cost treatments and supportive measures timely [41]. Zeng et al. identified several new proteomics biomarkers (SLC25A24, UBQLN1, and CREB3L3) and developed a practical nomogram using these for individualized monitoring of sepsis mortality risk, a valuable tool for promoting personalized treatment strategies [46]. Despite the model demonstrating significant potential for non-invasive omics in predicting patient-important outcomes, its clinical translation must be approached cautiously due to its small sample size, single-center retrospective design, and lack of independent external validation.

Proteomics has demonstrated enormous value in sepsis mechanism parsing, biomarker discovery, diagnosis and monitoring, and treatment guidance. Proteomics studies based on plasma and urine not only reveal molecular features of immunity, metabolism, and organ damage in sepsis but also provide key targets for developing precise diagnostic, monitoring tools, and targeted therapies. With ongoing technological innovations, proteomics is expected to further drive sepsis from “one-size-fits-all” treatment to individualized medicine, ultimately improving patient outcomes.

### 2.4. Metabolomics

Metabolomics studies metabolite changes post-internal/external perturbations. As downstream products of genes, transcription, and protein modifications, metabolites directly reflect physiological/pathological states and responses to stimuli. In sepsis, it reveals metabolic disorders, aiding early diagnosis, monitoring, prognosis, and therapeutic target identification, with broad prospects.

Studies have found that metabolomics plays an important role in early identification of high-risk sepsis populations. For trauma-induced sepsis, Gou et al. compared plasma metabolomes of patients developing sepsis after trauma (TDS) versus those not (TDDS), identifying 5 early predictive markers and 5 early diagnostic biomarkers [47]. These metabolite panels lay the foundation for future point-of-care diagnostic tools capable of predicting sepsis risk almost immediately following an injury. Schmerler et al. used plasma metabolomics and reported acylcarnitines and glycerophosphocholines as discriminative markers for sepsis, demonstrating metabolic differences between sepsis and non-infectious SIRS samples [48]. The stability and rapid detection capability of these small molecules give metabolomics a distinct advantage in the emergency setting for quickly resolving the major clinical dilemma of distinguishing infectious from non-infectious inflammation.

Wang et al., in a meta-analysis of 1287 individuals and 2509 metabolites, identified specific amino acids, mitochondrial metabolites, eicosanoids, and lyso-phospholipids as sepsis biomarkers, emphasizing the potential value of death-associated metabolic pathways as predictors of sepsis mortality [49]. Chang et al. identified distinct metabolic patterns between sepsis-induced ARDS patients and non-ARDS controls [50]. These metabolomics-based discoveries lay the foundation for future sepsis diagnosis and monitoring research, aiding early prevention, diagnosis, and treatment based on specific metabolites and pathways.

### 2.5. Multi-Omics Integration

**Figure 2:**
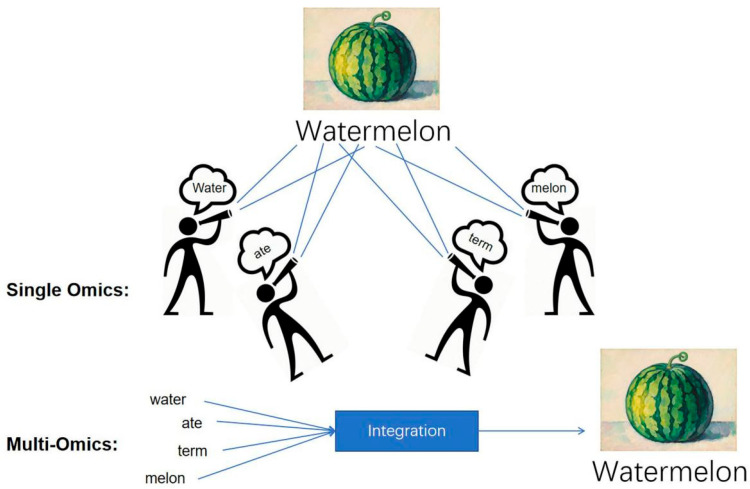
The Multi-Omics Advantage: Reconstructing a Holistic Molecular Portrait of Sepsis (The Watermelon Metaphor). This figure uses the metaphor of a fragmented “watermelon” to illustrate the necessity of data synergy in sepsis research. Single omics approaches are limited to observing disparate fragments, providing only a partial view and failing to grasp the complete, systemic nature of the disease. In contrast, the multi-omics approach integrates these multidimensional data points through AI/Bioinformatics and comprehensive analysis. This integration effectively reconstructs the complete “watermelon,” yielding a holistic and robust molecular portrait. This synergy is essential for overcoming the complex heterogeneity of sepsis, enabling deeper insights into pathophysiology, and ultimately facilitating more precise diagnosis and targeted treatment.

In recent years, multi-omics technologies have been integrated into sepsis research [51,52]. Compared to single omics capturing only one dimension of disease information, multi-omics integration achieves multi-dimensional dynamic parsing, significantly improving diagnostic precision and enhancing real-time monitoring of disease progression, thereby more systematically revealing the biological panorama of sepsis (Figure 2).

Huang et al. proposed a diagnostic model called IC3, composed of inosine, creatine, and 3-hydroxybutyrate, by integrating proteomics and metabolomics, which can assist in early screening of sepsis-associated AKI (SA-AKI) [53]. Through the integration of genomic, proteomic, and metabolomic data, Tong et al. systematically screened and validated 24 sepsis-associated genes, 6 genes associated with 28-day mortality, and 1 mediating metabolite using Mendelian randomization. Via drug prediction, molecular docking, and side-effect evaluation, multiple actionable targets (e.g., PDGFB, MRPL52) and potential therapeutics were identified. This study constitutes the first multi-omics research framework for sepsis, bridging genetic mechanisms to clinical intervention [54]. Our team integrated multi-omics data from 494 septic shock patients to develop a fluid therapy guidance model. Using LASSO regression, we constructed a “restrictive fluid benefit score” from transcriptomic data and translated it into a 6-protein biomarker panel detectable within 3–5 h. This approach achieved an 18% reduction in mortality risk when clinical practice aligned with model recommendations [55].

By integrating these data, researchers can better capture cellular heterogeneity and disease complexity. For example, the single-cell multiomics data clustering (scMDCL) uses a deep collaborative contrastive learning framework to integrate multiple omics data, fully utilizing intercellular relationships and interactions between different omics features, thereby effectively improving clustering performance of multi-omics data [56]. This framework not only achieves extraction and enhancement of cell features but also fully utilizes cell topological information through graph autoencoders and feature information enhancement modules, demonstrating the powerful potential of multi-omics integration. In tumor research, Multiview Clustering Method With Low-Rank (MVCLRS) uses low-rank and sparse constraints to construct similarity matrices, capturing local similarities in omics data, and then integrates multi-omics data to form a global consensus structure [57]. Additionally, the MOINER framework, through information enhancement and image representation learning, uses self-attention mechanisms to capture intrinsic associations between omics features, performing excellently in multi-omics early integration and biomarker discovery [58].

However, integrating omics data still faces significant difficulties, with data heterogeneity and the curse of dimensionality as major obstacles. First, data heterogeneity is reflected in differences between different omics data, which not only differ in technical generation but also in biological significance. For example, genomic data may involve gene mutations and copy number variations, while transcriptomic data focuses on gene expression levels. This diversity requires new algorithms and methods for effective integration [59,60]. Additionally, the curse of dimensionality in multi-omics data, with its high-dimensional nature, increases computational burden and complicates extracting meaningful information. Traditional statistical methods often struggle in such high-dimensional spaces, potentially leading to overfitting or information loss. Thus, advanced machine learning techniques like deep learning and graph neural networks are needed to improve integration efficiency and accuracy. Related studies show that deep learning models perform superiorly in handling multi-omics data, effectively capturing local and global structural information [61,62].

In addressing these challenges, data-driven and knowledge-guided integration strategies are widely applied. Data-driven methods rely on machine learning algorithms to automatically discover patterns, while knowledge-guided methods incorporate domain expert knowledge through network or pathway models for data integration. This combination not only improves integration accuracy but also enhances biological interpretability [59]. With the development of machine learning and AI technologies, parsing and integrating multi-omics data become increasingly efficient. Researchers use machine learning algorithms to mine potential biomarkers from multi-omics data and establish prognostic models, which can precisely assess sepsis patient prognosis in clinical settings [63].

## 3. Current Clinical Application Status of Omics Technologies in Sepsis Diagnosis

### 3.1. Combination of Traditional Biochemical and Immunological Indicators with Omics Biomarkers

In sepsis diagnosis and monitoring, traditional biochemical and immunological indicators are widely used but have limitations [64]. Existing biomarkers such as CRP, PCT, and white blood cell count have certain reference value in clinical practice but often lack sufficient sensitivity and specificity, leading to insufficient accuracy in early diagnosis and patient stratification. For example, elevated CRP may not be specific to sepsis and can result in false positives due to other inflammatory or infectious diseases [65]. Moreover, these traditional indicators typically cannot comprehensively reflect patients’ immune status and pathophysiological changes, making it difficult for doctors to make rapid and accurate decisions in complex clinical environments.

At the same time, the dynamic changes in traditional biomarkers are difficult to monitor in real-time, limiting their effectiveness in clinical application. Sepsis progresses rapidly, with patients potentially experiencing significant physiological changes in a short time; relying on single biomarkers for monitoring may miss timely intervention opportunities. The review by Pierrakos et al. also highlights that, although a substantial number of biomarkers (approximately 200 according to existing literature) have been proposed in the field of sepsis, most traditional biomarkers still exhibit significant limitations in terms of clinical translation and their value in guiding treatment. These limitations primarily stem from a lack of specificity (elevation also occurs in non-infectious inflammatory conditions), a unidimensional nature (inability to capture the dynamic and multi-systemic pathological changes of the disease), and most critically, an inability to address the high degree of clinical and molecular heterogeneity in sepsis [14]. This heterogeneity is a major reason for the overall failure of drug clinical trials, rendering “one-size-fits-all” treatment strategies largely ineffective.

In summary, traditional biochemical and immunological indicators have significant limitations in sepsis management, urgently needing combination with more comprehensive omics biomarkers to enhance diagnostic accuracy and treatment targeting [66].

In recent years, the development of omics technologies has provided new perspectives and methods for sepsis diagnosis [67]. Omics biomarkers, such as those from genomics, transcriptomics, proteomics, and metabolomics, through integrated analysis of multi-level biological data, can effectively improve diagnostic sensitivity and specificity. For example, studies show that integrating different levels of data can identify differentially expressed mature and immature neutrophil subsets in sepsis patients, closely related to changes in multiple proteins, metabolites, and lipids [68]. Omics biomarkers also have the advantage of dynamic monitoring, able to real-time reflect patients’ pathophysiological states. For example, metabolomics analysis can identify metabolite changes associated with sepsis, often appearing before changes in traditional biochemical indicators, providing more sensitive early warning signals for clinical use. Additionally, weighted linear regression analysis of multi-omics data can construct more precise statistical models for sepsis biomarker identification and clinical application.

Furthermore, the multi-dimensional and mechanistic information provided by omics biomarkers enables the identification of molecular endotypes with distinct prognostic patterns. This elevates omics from a predictive tool to a therapeutic guide, offering for the first time the possibility of directing targeted immunomodulatory or metabolic support therapies. This represents a unique advantage of omics approaches. Regarding diagnostic claims, we acknowledge that many omics studies in the discovery phase still rely on clinical diagnostic criteria such as Sepsis-3.0 as a reference—a recognized limitation. Future research must shift its focus from “diagnostic alignment” to “improving patient-important outcomes”. The ultimate validation goal for the integration of omics biomarkers and AI is to demonstrate that treatment strategies guided by them can significantly improve 28-day/90-day mortality or organ function recovery time, thereby establishing an independent clinical value standard that surpasses reliance on clinical diagnostic references. Subsequent chapters of this review will elaborate in detail on how current research across different omics fields is working to concretely achieve this precision management objective by discovering molecular endotypes and functional state indicators.

In summary, the introduction of omics biomarkers not only overcomes the limitations of traditional biochemical and immunological indicators but also provides valuable tools for early sepsis diagnosis and precision medicine. Future research should continue to explore their application potential in clinical practice to improve sepsis patient prognosis and survival rates.

### 3.2. Development of Bedside Rapid Diagnostic Tools Driven by Omics Technologies

The rapid development of omics technologies provides new opportunities for developing bedside rapid diagnostic tools for sepsis [67]. High-throughput detection platforms based on genomics, transcriptomics, proteomics, and metabolomics enable simultaneous analysis of numerous biomarkers, facilitating early identification and monitoring of sepsis. For example, a systematic review shows that omics-based detection in neonatal sepsis diagnosis exhibits up to 88% sensitivity and 76% specificity, indicating these technologies can significantly improve diagnostic accuracy [68]. One study developed a microfluidic digital ELISA platform, first achieving high temporal resolution multi-cytokine real-time monitoring in small animal models with only 3.5 μL whole blood. Validated in a mouse sepsis model, the platform completes detection in 2 h and predicts liver injury 24 h later through early cytokine levels, significantly reducing animal usage [69]. Advances in point-of-care technologies (such as biosensors and nanotechnology) enable rapid bedside analysis of whole blood samples, shortening the time required for traditional cultures and securing critical windows for early intervention in acute conditions like sepsis [70,71]. The emergence of portable detection platforms has also revolutionized sepsis monitoring. These devices typically integrate multiple sensors to collect physiological parameters like heart rate, respiratory rate, and temperature in real-time, transmitting data wirelessly to clinicians. For example, a soft, wearable wound dressing system can real-time monitor wound conditions and sepsis-related biomarkers and achieve wireless data transmission via near-field communication, greatly facilitating clinical use [72]. By integrating these high-throughput and real-time monitoring technologies, clinicians can quickly obtain biomarker information for sepsis patients, effectively guiding treatment plans and improving outcomes.

**Figure 3:**
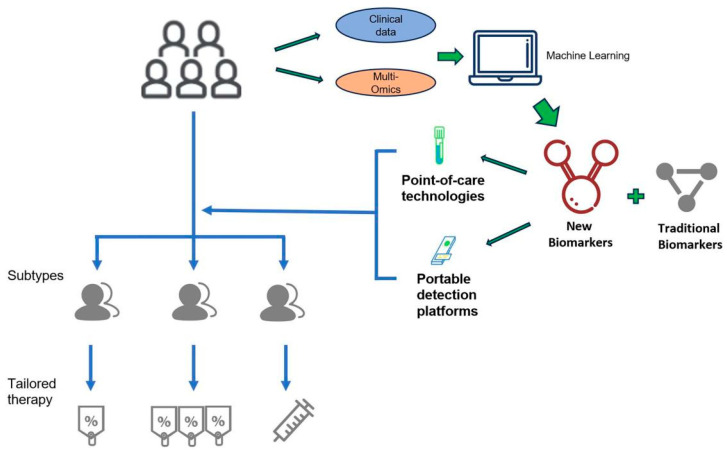
Future Vision of Omics-Driven Precision Management at the Bedside. This figure presents an idealized scenario where omics and AI technologies, having overcome current Turnaround Time barriers, are implemented at the ICU bedside. It highlights a future closed-loop system realized through rapid, portable omics devices for real-time sample analysis, combined with AI systems to output dynamic molecular monitoring and real-time clinical decision support. This serves as a translational roadmap, guiding future technology development and clinical trials necessary to achieve dynamic, personalized, and real-time intervention for the disease.

Single biomarkers lack comprehensive diagnostic value in sepsis; multi-biomarker models are crucial. Integrating omics data identifies sepsis-specific combinations that better reflect patient status. For example, one study used time-dependent multi-omics integration to reveal the role of the TLR4 pathway in sepsis-associated liver dysfunction models, surpassing limitations of single omics analysis and uncovering potential sepsis pathological mechanisms [73]. Additionally, combining machine learning and data mining techniques allows deeper analysis of multi-omics data; for instance, algorithmic models can predict patient responses to specific treatments, achieving personalized medicine. Such multi-biomarker joint diagnostic models not only improve early sepsis identification rates but also provide important bases for individualized treatment, aiding clinical management and patient outcomes (Figure 3).

Through these technological applications, sepsis diagnosis and monitoring are advancing toward greater precision and efficiency. Future research should continue exploring how to further optimize these tools’ clinical applications to improve sepsis patient survival rates and quality of life.

## 4. Application of Omics in Sepsis Monitoring and Prognosis Assessment

### 4.1. Dynamic Monitoring of Immune Function and Inflammatory Status

Dynamic monitoring of immune function and inflammation is crucial in sepsis management. Omics technologies reveal immune cell phenotypic changes and dysfunction, key drivers of disease progression. For example, studies show a significant increase in myeloid-derived suppressor cells (MDSCs) in sepsis patients, which suppress T cell function and promote immunosuppressive states [74]. Additionally, activation states of monocytes and lymphocytes can be assessed through detection of cell surface markers, helping evaluate patients’ immune status and response to infection, providing potential biomarkers for clinical use [75]. Notably, dynamic changes in cytokines and metabolites can reflect immune cell activity. In sepsis patients, inflammatory factors like TNF-α and IL-6 are often significantly elevated, participating in inflammatory responses and potentially affecting immune cell functions [76]. Monitoring cytokines can provide important prognostic information for clinical use, aiding personalized treatment schemes. Furthermore, metabolite changes like lactate and fatty acids are closely related to immune cell functions; dynamic monitoring of these metabolites helps further understand sepsis pathophysiological mechanisms.

In summary, omics-based analysis of immune cell phenotypes and functions not only provides new ideas for early sepsis diagnosis but also offers scientific bases for targeted immunomodulatory treatment strategies.

### 4.2. Construction of Prognostic Prediction Models Using Omics

In sepsis prognosis prediction, omics and multi-omics data integration provides new perspectives and methods for risk stratification. For example, changes in certain metabolites are closely related to sepsis severity and prognosis, providing bases for personalized treatment [77]. Simultaneously, joint analysis of proteomics and metabolomics can reveal complex metabolic pathways in sepsis patients, identifying potential biomarkers and supporting personalized treatment schemes [78].

Machine learning and statistical models are becoming increasingly important in sepsis prognosis prediction; machine learning models can handle large heterogeneous data and identify complex nonlinear relationships. These methods effectively analyze complex clinical and multi-omics data, identifying key features and patterns related to disease progression [79,80]. Integrating information from multiple biomarkers and identifying key molecules associated with sepsis, these models can more accurately predict disease course and prognosis, providing support for physicians [81]. This not only helps physicians better understand disease progression but also provides stronger bases for clinical decisions [82].

With technological advances, real-time monitoring and dynamic model adjustment capabilities are continuously improving. By integrating multi-omics data with advanced analytical techniques, machine learning-based prognostic prediction models are expected to increasingly integrate into clinical workflows. This will achieve personalized medicine goals and provide more precise prevention and treatment measures for sepsis patients, thereby laying the foundation for improving overall patient management levels.

## 5. Application of Artificial Intelligence and Omics Data Fusion in Sepsis: Challenges and Future Prospects

### 5.1. AI-Assisted Omics Data Analysis and Clinical Decision Support

**Figure 4:**
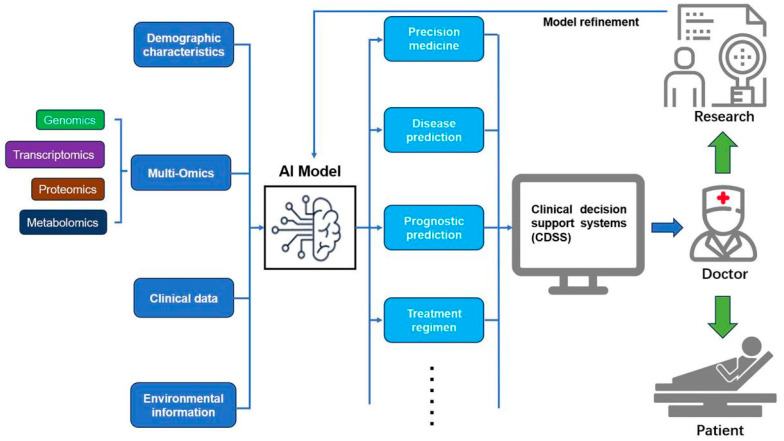
This schematic illustrates the application of artificial intelligence (AI) and multi-omics data fusion in sepsis management. The AI model integrates multi-omics data (genomics, transcriptomics, proteomics, metabolomics), demographic characteristics, clinical data, and environmental information to enable precision medicine, disease prediction, prognostic prediction, and treatment regimen optimization. The output is channeled through clinical decision support systems (CDSSs), assisting doctors in delivering personalized care to patients. Continuous model refinement, driven by research feedback, enhances the system’s accuracy and effectiveness in improving patient outcomes.

Artificial intelligence (AI) integrated with multi-omics data (genomics, transcriptomics, proteomics, metabolomics) enables feature extraction, pattern recognition, and dynamic monitoring of immune status in sepsis [83]. By processing vast datasets from electronic health records (EHRs) and omics layers, AI identifies biomarkers, predicts disease trends, and supports personalized treatment [84]. Systems like SepsisLab exemplify AI-driven clinical decision support (CDSS), enhancing early diagnosis, risk stratification, and real-time treatment adjustment while reducing decision uncertainty [85,86,87,88]. Deep workflow integration improves clinician trust and outcome optimization [89,90]. In summary, AI-omics fusion provides powerful tools for precision sepsis management, improving diagnostic accuracy and survival rates (Figure 4).

Currently, most omics-based AI models remain at the retrospective discovery stage; their clinical reliability urgently needs to be demonstrated through independent prospective or external validation. The value of a CDSS should not be limited to predictive metrics (such as AUC), but must be measured by its quantified impact on patient-important outcomes (e.g., percentage reduction in mortality, increase in organ support-free days) or workflow optimization (e.g., reduced decision-making time).

### 5.2. Bottlenecks in Technology, Data Analysis, and Clinical Translation

Data heterogeneity, non-standardized workflows, and format inconsistencies complicate multi-omics integration and comparison [91,92]. Combining omics with clinical data further increases the technical burden [93]. High-dimensional data suffer from the “curse of dimensionality,” noise, and biological interpretability challenges, exacerbated by individual variability and treatment responses [94]. Although machine learning and integration strategies (e.g., dimensionality reduction) offer solutions [51,83], the path to clinical adoption encounters specific real-world barriers that transcend mere data complexity.

Firstly, there is the crucial issue of Time-to-Result (TAT). Sepsis is a time-sensitive emergency requiring intervention within the “golden hour.” However, most high-throughput omics platforms (such as mass spectrometry and next-generation sequencing) necessitate lengthy sample preparation, run times, and bioinformatics analysis, resulting in a TAT that often exceeds the critical decision-making window. The current lack of real-time feasibility for complex omics analysis remains a significant technical barrier to bedside application.

Secondly, the economic and resource burden is substantial. Clinical translation requires not only large-scale validation and infrastructure expansion [95] but also mandates overcoming the high operating costs of dedicated equipment and reagents, as well as the continuous demand for specialized bioinformatics expertise. Resource-limited settings face acute barriers in equipment accessibility, expertise, and policy support, creating a significant economic barrier to wide-scale adoption. Furthermore, the existing challenges of data heterogeneity are compounded by the lack of widely accepted multi-center standardization protocols for the pre-analytical phase, which limits reproducibility and generalization across diverse patient populations.

### 5.3. Ethical Considerations

Omics research involves sensitive patient data, mandating strict privacy protection, informed consent, and ethical oversight to comply with legal standards [95,96]. Anonymization, secure storage, and transparent data-use policies are essential to balance scientific progress with patient rights. Public education is critical to enhance acceptance and minimize ethical disputes.

### 5.4. Future Development Trends

Sepsis research will advance through deep multi-omics integration, single-cell omics, and time-dependent analysis (TDMI) to uncover pathophysiology and novel biomarkers. AI-enabled patient subtyping will drive individualized precision treatment, overcoming “one-size-fits-all” limitations [83,97,98]. Emerging technologies like spatial omics and AI fusion will elucidate cellular interactions and predict responses, accelerating targeted therapies and clinical transformation [99,100].

However, the realization of clinical value from these cutting-edge technologies and discoveries must adhere to a rigorous translational evaluation framework to ensure they ultimately improve patient-important outcomes. Future research urgently needs to shift from retrospective exploration to prospective, multicenter, randomized controlled trials (RCTs). When evaluating omics-driven biomarkers or AI models, the study design must clearly define the following: the control group should be set as the current Standard of Care (SOC); primary endpoints must focus on Patient-Important Outcomes, including 28-day/90-day all-cause mortality and organ support-free days; the ultimate success criterion should be demonstrating that omics-guided personalized interventions can achieve statistically significant and clinically meaningful improvements in these primary endpoints compared to SOC.

For AI-assisted Clinical Decision Support Systems (CDSSs), success criteria should additionally include demonstrating the ability to reduce decision-making time for critical interventions or improve treatment adherence, while ensuring no increase in adverse events. Well-defined translational pathways and quantified success criteria represent the final and most crucial step in ensuring omics and AI technologies transition from bench to bedside.

## 6. Collaborative Data Ecosystems and Biorepositories

Large-scale, multi-institutional collaboration is essential to overcome the challenges of sepsis heterogeneity and limited single-center sample sizes. Collaborative data ecosystems and biorepositories are indispensable for advancing multi-omics research by pooling diverse patient data and biological samples.

For instance, the CMAISE (Chinese Multi-Omics Advances in Sepsis) database serves as an impressive example of this living collaboration, integrating clinical, genomic, transcriptomic, proteomic, and metabolomic data from multiple centers across China. CMAISE-v1.0 was officially opened globally, providing longitudinal blood samples and clinical phenotypes from days 1, 3, and 5 for sepsis and septic shock patients, including complex complications like AKI and ARDS. By standardizing data collection and sharing, this model is crucial for accelerating the discovery and validation of robust, generalized sepsis endotypes and biomarkers that are not susceptible to single-center bias. CMAISE data has yielded key publications in top journals: proteomics identified 6 protein markers (AUC = 0.802) guiding restrictive fluids to reduce septic shock mortality from 16% to 13% [55]; transcriptomics enabled dynamic trajectory prediction of sepsis-AKI for early renal protection [101]; scRNA-seq revealed neutrophil heterogeneity and prognostic subsets for targeted immunomodulation [102]; and “disease axis” continuous subtyping optimized precision treatment [103]. CMAISE bridges multi-omics discoveries to emergency/critical care practice.

## 7. Conclusions

Multi-omics technologies are shifting sepsis diagnosis and monitoring from “symptom-driven” to “molecule-driven”: genomics locks susceptibility loci, transcriptomics signals infection-inflammation via peripheral blood gene expression profiles before symptoms appear, proteomics tracks dynamic changes in inflammation, coagulation, and organ damage-related proteins in real-time, and metabolomics continuously quantifies disease progression using key metabolites like lactate and phenylalanine. The central thesis of this review is to demonstrate how omics and AI jointly tackle sepsis heterogeneity—encompassing pathogen diversity, host immune variability, and dynamic organ injury—thereby enabling a transition from uniform protocols to precision and personalized management. Integrating these levels along a timeline not only distinguishes infection from sterile inflammation with high sensitivity and specificity, and predicts organ dysfunction risk, but also completes patient subtyping within hours, providing an “operable molecular dashboard” for bedside rapid decisions.

AI’s involvement further amplifies this advantage: machine learning models automatically select the most diagnostically/prognostically valuable combinations from massive omics features, constructing real-time updatable digital early warning systems, enabling individualized risk scores and treatment target prompts early in admission. Despite needs for data standardization, clinical validation, and ethical frameworks, “multi-omics + AI” has transformed sepsis monitoring from passive observation to active prediction, significantly compressing the time window from symptom onset to precise intervention, laying feasible technical foundations for true real-time monitoring and personalized treatment.

## Data Availability

No new data were created or analyzed in this study.
